# Making the COVID-19 Pandemic a Driver for Digital Health: Brazilian Strategies

**DOI:** 10.2196/28643

**Published:** 2021-06-29

**Authors:** Bruna Donida, Cristiano André da Costa, Juliana Nichterwitz Scherer

**Affiliations:** 1 SOFTWARELAB - Software Innovation Laboratory Universidade do Vale do Rio dos Sinos São Leopoldo Brazil

**Keywords:** COVID-19, digital technology, Brazil, public health, medical informatics, digital health, strategy, outbreak, system, data, health data, implementation, monitoring

## Abstract

The COVID-19 outbreak exposed several problems faced by health systems worldwide, especially concerning the safe and rapid generation and sharing of health data. However, this pandemic scenario has also facilitated the rapid implementation and monitoring of technologies in the health field. In view of the occurrence of the public emergency caused by SARS-CoV-2 in Brazil, the Department of Informatics of the Brazilian Unified Health System created a contingency plan. In this paper, we aim to report the digital health strategies applied in Brazil and the first results obtained during the fight against COVID-19. Conecte SUS, a platform created to store all the health data of an individual throughout their life, is the center point of the Brazilian digital strategy. Access to the platform can be obtained through an app by the patient and the health professionals involved in the case. Health data sharing became possible due to the creation of the National Health Data Network (*Rede Nacional de Dados em Saúde*, RNDS). A mobile app was developed to guide citizens regarding the need to go to a health facility and to assist in disseminating official news about the virus. The mobile app can also alert the user if they have had contact with an infected person. The official numbers of cases and available hospital beds are updated and published daily on a website containing interactive graphs. These data are obtained due to creating a web-based notification system that uses the RNDS to share information about the cases. Preclinical care through telemedicine has become essential to prevent overload in health facilities. The exchange of experiences between medical teams from large centers and small hospitals was made possible using telehealth. Brazil took a giant step toward digital health adoption, creating and implementing important initiatives; however, these initiatives do not yet cover the entire health system. It is expected that the sharing of health data that are maintained and authorized by the patient will become a reality in the near future. The intention is to obtain better clinical outcomes, cost reduction, and faster and better services in the public health network.

## Introduction

The year 2020 was marked by the outbreak of COVID-19, the disease caused by the highly contagious virus SARS-CoV-2, which created many challenges for the scientific community and health services worldwide. COVID-19 was declared a pandemic by the World Health Organization (WHO) on March 11, forcing public health authorities in all countries to adopt measures focusing on surveillance, rapid case identification, interruption of community transmission, and strong public communication to contain the spread of the virus, mitigate its impact on human health, and attempt to prevent the collapse of health systems [1]. In this context, effective, integrated, and safe recording, management, and follow-up of patients’ clinical data at the different levels of a health system are fundamental to better address the situation imposed by the COVID-19 pandemic [2,3]. However, health care systems are composed of multiple agents and services, which cannot always share patients’ clinical data adequately and at the necessary speed to address the pandemic scenario.

The COVID-19 pandemic has several peculiar characteristics that set it apart from other pandemics previously faced by the world, such as the number of infected individuals, the high transmissibility levels, the broad spectrum of symptoms, and the rapid evolution of patients to severe conditions [4]. In addition, the COVID-19 pandemic is occurring in an era of massive technological advancement, when digital health solutions have been extensively discussed but have not yet been widely deployed and accepted [5]. Considering this adverse scenario, at the same time that the COVID-19 pandemic has exposed the deficiencies of health care systems worldwide, it is providing an opportunity to develop and test innovative solutions extremely quickly to strengthen public health measures [6]. In this context, in this paper, we aim to share the Brazilian digital health initiatives that were implemented to mitigate the damage caused by COVID-19. These initiatives were created by a special committee linked to the information technology (IT) department of the Brazilian Unified Health System, which is considered to be one of the most extensive public health systems in the world.

## The Brazilian Health System

On February 3, 2020, Brazil declared a public health emergency of national importance. The first case of COVID-19 in Brazil was confirmed in São Paulo on February 26 [7], two months after China notified the WHO about the emergence of a series of cases of pneumonia of unknown cause [8]. As of May 22, 2021, Brazil had an accumulated record of 16,047,439 cases and 448,208 deaths, with the number of cases increasing every day [9].

The dynamic and high-risk scenario for the population caused by the new coronavirus required forceful responses from the entire health system, especially from the Unified Health System (*Sistema Único de Saúde*, SUS). SUS is the Brazilian public health system; it was created in 1988, inspired by the United Kingdom's National Health Service. To date, Brazil is considered to be the only country with a population of more than 200 million people to have a universal health care system, and approximately 75% of the population uses SUS exclusively. SUS coordinates national actions and orchestrates the efforts of states, municipalities, and even supplementary health; it is maintained by public power, with supplementary participation of private initiatives [10].

Although the use of information and communications technology (ICT) in the health area was guaranteed in Brazil by Organic Law No 8080 in 1990, due to the lack of investments and ethical and bureaucratic issues, the insertion of technology and data sharing has not yet become a reality for health services. Based on the National eHealth Strategy Toolkit published by the WHO in 2012, the Brazilian digital health strategy was approved in 2017 and defined the digital health strategy as a fundamental SUS dimension. The program that “materializes” the Brazilian eHealth strategy is Conecte SUS, which is based on two structuring projects: the National Health Data Network (*Rede Nacional de Dados em Saúde*, RNDS) and the Program to Support Computerization and Qualification of Primary Health Care Data (*Programa de Apoio à Informatização e Qualificação dos Dados da Atenção Primária à Saúde*, Informatiza APS). The RNDS aims to promote the exchange of information between the different services of the Health Care Network, allowing transition and continuity of care in the public and private sectors. Informatiza APS aims to support the computerization of health units and the qualification of primary health care data across the country [11].

**Figure 1:**
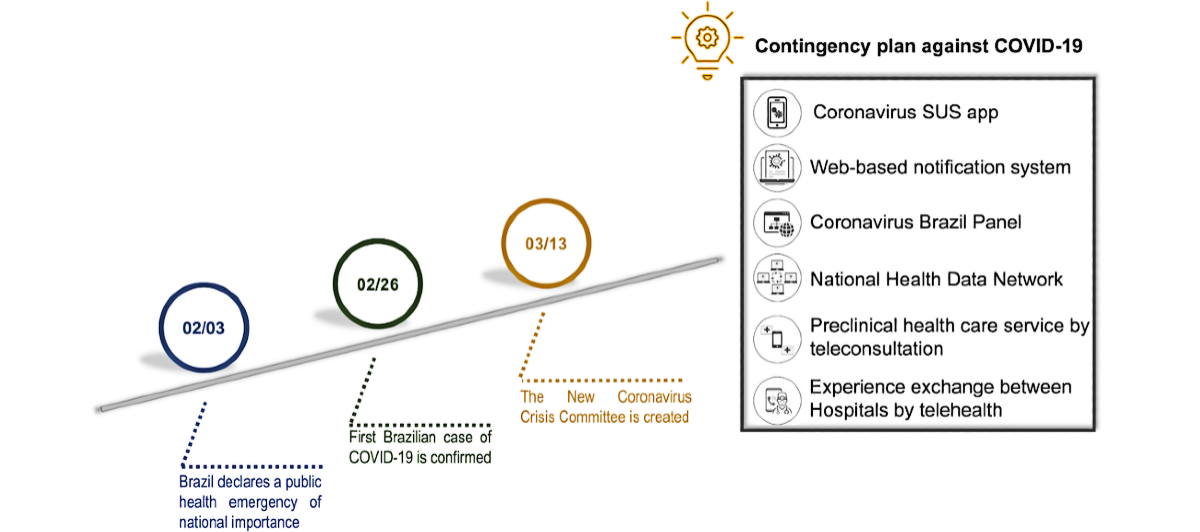
The digital health strategies deployed in Brazil in response to COVID-19. SUS: Sistema Único de Saúde (Unified Health System).

Conecte SUS is a standardized, modern, and interoperable platform of services, information, and connectivity that is, in itself, transformative for health. This platform predicts the integration of citizens’ health information in an extensive data network organized by the Ministry of Health (RNDS); this platform will bring benefits both to citizens—who will have access to their trajectory in SUS—and to health professionals and managers, who will have a set of information that will improve the continuity of care and decision-making [11]. Conecte SUS was structured as a pilot project. In November 2019, this project started in one Brazilian state to validate the conducted planning and refine the proposal to expand the program throughout Brazil. However, on March 2020, plans designed within the Brazilian strategy for digital health faced the COVID-19 pandemic. On March 13, 2020, the SUS IT department (DATASUS) established the New Coronavirus Crisis Committee, which is responsible for evaluating new health management technologies and prioritizing the care and prevention guidelines of the Ministry of Health itself. The main strategies and actions adopted by DATASUS to assist the Ministry of Health were published as a contingency plan. The contingency plan proposed a series of strategies for a quick and efficient response to the virus through direct communication with the population and with public and private health systems. Thus, through the contingency plan, Brazilian strategies for digital health transformation, such as Conecte SUS and the RNDS, were redirected to address the virus. Moreover, the contingency plan developed some specific strategies for the pandemic moment: the creation of an app related to the virus (Coronavirus SUS app), the restructuring of a compulsory web-based notification system, a web-based panel of official disease data, and the use of telemedicine for patient care (Figure 1). All these strategies will be explained in the next section.

## Contingency Plan

Addressing the COVID-19 pandemic requires information at different levels, from the registration of notifications, deaths, and results of performed tests to the provision of services aimed at prevention and care. Among these services are self-assessment, teleconsultation, active search for patients, and advanced applications for identifying trends and vulnerable populations. To encompass all these demands, Conecte SUS systematizes a health care and data ecosystem for COVID-19. This ecosystem comprises specific layers for information security, interoperability between systems, notification processes and health surveillance, mobile apps, and access channels, as detailed below.

The RNDS is a federated interoperability layer. Several digital health applications, notably electronic health records (EHRs), hospital and laboratory management systems, portals, and mobile apps (for citizens, health professionals, and managers), exchange information through a service bus. The RNDS had recently been tested as a pilot project in a Brazilian state when the COVID-19 pandemic arrived in Brazil. As health emergency care priorities were modified, the RNDS had to quickly assume the COVID-19 national data repository position, acting on reception and integration of case notifications and results of laboratory tests as well as distribution and sharing of data and epidemiological information. The data about the epidemiologic situation of COVID-19 in Brazil were published in the Coronavirus Brazil Panel, the official communication vehicle. The Ministry of Health provides daily updates of the number of confirmed cases of COVID-19, the number of deaths, and the lethality rate of the virus based on data provided by the state health departments of the 27 Brazilian federative units. The website presents data related to the country, separate data for each state, and cases per epidemiological week as well as an epidemic curve. This communication platform contains georeferenced records of all COVID-19 cases registered in the country. The platform provides an interactive, graphic view of the cases [12].

In its first phase, the RNDS allowed the sharing of COVID-19 laboratory test results performed anywhere in the country through services developed according to the HL7 Fast Healthcare Interoperability (FHIR) standards and Logical Observation Identifiers Names and Codes terminology. Currently, the RNDS is also integrated with the web-based notification system, allowing interoperability between the reported cases and the results of laboratory tests. In this context, for cases registered in the network, the test results are received automatically, and tests performed on individuals who have not yet been informed generate a notification. Interoperability allows digital automation of the process, which was previously conducted manually by epidemiological surveillance teams and clinical laboratories. By the end of April 2021, more than 14 million results of COVID-19 tests were sent to the RNDS by 153 laboratories [13].

Additionally, the Conecte SUS Portal is being made available, where citizens, health professionals, and managers will be able to access information in the RNDS with the primary purpose of improving health care and allowing continuity of care. In March 2021, the Conecte SUS app was endowed with new features to facilitate the vaccination process in the country: the Digital Vaccination Card and the National Vaccination Certificate for anyone who is immunized against COVID-19. In this context, for all citizens to access their information, states and municipalities must send information from the Administrated Immunobiological Registry to the RNDS. Up to April 2021, more than 39 million vaccine registrations were sent to the RNDS, and the Conecte SUS mobile app was downloaded more than 11 million times [13].

COVID-19 case control in Brazil started on March 20, 2021, when the Ministry of Health declared the community transmission stage of COVID-19 and determined mandatory immediate notification (within 24 hours) of all suspected cases—including cases of influenza and severe acute respiratory syndrome (SARS)—for public and private services [14]. There are two types of notification systems: one for mild influenza, mainly used by basic health units, called e-SUS Notifica, and another, used to record hospitalized SARS cases and deaths, called Sistema de Informação de Vigilância Epidemiológica da Gripe (SIVEP-Gripe).

The notification system, e-SUS Notifica, was exclusively improved to update COVID-19 and receive daily data from each basic health unit in the whole country and private health care units. Consolidated accounts are created, and the numbers of individuals with suspected infection, individuals with confirmed disease, and deaths are automatically obtained. This total number is updated every day at 7 PM on the Ministry of Health website if everything proceeds as expected. In practice, the system is fed from a consolidated number. Every day, workers assigned to each function update their unit’s data and summarize the numbers that form the consolidated report released by the Ministry. These workers, who are often physicians or a nurses, reconcile assistance with notification. This notification process is already part of the work routine in health services and is governed by Ordinance 204/2016, which lists the diseases and conditions of compulsory notification. The factor that has changed from the previous daily process to that of the COVID-19 pandemic is the time of notification. As the pandemic is a public health event that has national and international importance, agility in treating the disease has exponential significance.

In addition to the notification, all patient information must be recorded in citizens’ medical records, preferably in an electronic version (EHR), to enable longitudinal and coordination of care as well as eventual epidemiological investigation and the subsequent formulation of policies and strategies for prevention. The registration must be performed directly by the professional who addressed the case and not simply by surveillance; moreover, confirmed cases should not be the only cases entered into the system. The judgment to define a suspected case must be clinical-epidemiological and performed by a health care professional. Upon laboratory confirmation, and through the interoperability allowed by the RNDS, the laboratory result is automatically inserted in the notification form.

To make the population aware of the disease caused by the new coronavirus and to assist in the dissemination of information for prevention and guidance, the Ministry of Health launched the Coronavirus-SUS app, which has the following features: list of the symptoms of COVID-19, advice on how to prevent the disease, actions to take in case of suspected infection, a map indicating nearby health units, and official Ministry of Health news focused on COVID-19. The app allows the user to assess their health status concerning COVID-19, performs automatic notifications based on health data entered by the user, and offers guidelines and recommendations for the user.

If necessary, the app directs the user to a teleconsultation or face-to-face clinical care. The latest update to the app provided contact tracing functionality, which generates a warning if the user has physically approached someone who tested positive for SARS-CoV-2 in the previous 14 days. The system depends on the voluntary collaboration of people who tested positive. Still, before generating the alert, this information is confirmed by cross-checking between the person’s examination and the integrated records of the surveillance platform (e-SUS Notifica) and the RNDS. The cell phones of the people who had contact (either acquaintances or strangers on the street) anonymously exchange keys via Bluetooth through the app. These keys are stored, and if, in the future, the owners of the keys test positive, all other users with whom they had contact will be notified. The app’s home page has a red button with the question “Are you feeling bad?” that brings up a list of questions to aid a self-diagnosis of COVID-19 infection. The app was made available in 10 countries, including North Korea, Panama, China, and Argentina, and it has already been downloaded by more than 10 million users [15].

One of the strategies developed by the Primary Health Care Secretary, in partnership with DATASUS, was the system of Preclinical Health Care–TeleSUS. In the call center/teleconsultation model, through four service channels (the Coronavirus SUS app, WhatsApp, Dial 136, and the Virtual Assistant on the Ministry of Health Portal), citizens can be evaluated, be notified, and receive a medical certificate, if necessary. The channels assist the patients through the ChatBot Service, Audible Recognition Unit Service, Preclinical Service, and remote monitoring. The TeleSUS initiative aims to promote home isolation of the potentially contaminated population or members of risk groups (those who do not show signs of severe disease), avoiding overcrowding in primary health care units. One of the structuring solutions of this system is a robot that makes telephone calls to citizens over 60 years of age to offer guidance, provide systematic follow-up, and, if necessary, refer them to a teleconsultation or face-to-face service. With the integration with the RNDS and e-SUS Notifica, the Preclinical Care System allows professionals to generate notifications related to the pandemic, access test results from public and private laboratories, and consult the patient’s clinical history through the Conecte SUS platform [16].

Another resource developed by the Ministry of Health, in partnership with the Institutional Development Support Program of the Unified Health System (*Programa de Apoio ao Desenvolvimento Institucional do Sistema Único de Saúde*, PROADI-SUS), offers, through the Tele-UTI Project COVID-19 Brazil, a daily routine/horizontal visit service using telemedicine resources, through which the multidisciplinary teams of large centers advise teams at smaller hospitals by teleconference. A hotline is also available for health professionals to assist in handling severe cases and discussing safety protocols every day from 7 AM to 7 PM.

## Challenges and Opportunities

The whole context of health data sharing becomes even stronger amid a pandemic, which requires daily updates of epidemiological data for control and decision-making by governments and health systems. The COVID-19 pandemic has demonstrated the importance and usefulness of digital health strategies and has allowed the insertion of these solutions into health care systems in the long term. However, it is essential to understand that digital health adoption is only in the early stages, not only in Brazil but worldwide [17]. Policy makers are first dealing with the considerable challenge of adapting technology to their domestic health frameworks. Furthermore, each country must consider the diversity and necessity of their population to increase the acceptability of digital technologies in health.

The need for urgency in decision-making implied by the COVID-19 outbreak required Brazil to take an important step toward digital health implementation. However, there is still a long way to go to fully implement digital solutions in the health area. One of the significant challenges for eHealth in Brazil is the computerization of primary care units. To enable the RNDS to share health data, health systems must have access to the internet and use the EHR. To achieve this, one of the pillars of the Conecte SUS program is Informatiza APS. The objective of Informatiza APS is to qualify health data and computerize all family health and primary health care teams in the country. Through the Informatiza APS project, between October 2019 and January 2021, the number of computerized units increased from 55% to 67% [13]. Additionally, over 44% of computerized units use EHR systems that are different from that created by the Ministry of Health (the Electronic Citizen Medical Record), generating losses in terms of data interoperability and integration between the different levels of the health system [11]. The main challenge raised by municipal managers for the advancement of computerization is the scarcity of infrastructure and trained teams to implement the EHR and guarantee data submission through the RNDS. Additional difficulties have been mentioned, such as power fluctuation (peaks and lack), internet connection (speed oscillation and lack of provider), public insecurity in units (theft of equipment), insufficiency of equipment, and lack of a deployment team [11].

Direct digitization at the point of data collection and automated reporting is not a reality worldwide. When we examine the COVID-19 cases and deaths reported in the United States, for example, the numbers drop substantially every Sunday and Monday, with case numbers rebounding later in the week. This fluctuation occurs because most countries still collect data through paper reporting and forms [18]. The use of web-based notifications directly at the point of care integrated with the RNDS allowed the Brazilian government to track COVID-19 more effectively. However, delays still occur in correct notification because some health units are not computerized and provide the notification manually.

In the current state of emergency, the Brazilian federal government enacted Law No 13989 of April 15, 2020, authorizing the use of telemedicine during the COVID-19 pandemic and allowing physicians to care for their patients virtually. In less than four months, TeleSUS made 7.4 million calls. Worldwide, telehealth will provide citizens with access to adequate and qualified information, and it represents an important alternative to avoid contagion and facilitate social distancing [19,20]. Telemedicine still faces barriers to its expansion due to the gaps and inequalities in access to ICT by health facilities and citizens; however, it can be used to care for patients who do not have COVID-19 [21]. Clinical trials have demonstrated that teleconsultations resulted in high satisfaction among health care providers and patients, independent of disease progression, with lower costs than traditional visits [22].

For the digital transformation to occur, several points must be discussed, among them the guarantee of equity and universality of access (premises of SUS) and ways to engage the population, health professionals, and managers. This is because individuals who do not have the necessary knowledge to use the technologies cannot benefit them [23]. Especially in a continental country such as Brazil, which has very marked social differences among its population, it is necessary to think about public policies for the insertion of technology in health that do not reinforce social inequality and do not result in worse outcomes for the most vulnerable population. Crawford and Serhal (2021) [24] proposed a Digital Health Equity Framework to consider the health equity factors. They point out that together with person-centered care, digital health equity should be incorporated into health provider training and should be supported at the individual, institutional, and social levels.

The development and use of apps in the health care field is a reality, and the number of mobile apps created has been increasing. These apps have been implemented for training, information sharing, risk assessment, self-management of symptoms, contact tracing, home monitoring, and decision making. They are considered valuable tools for citizens, health professionals, and decision-makers in facing critical challenges imposed by the pandemic. In a general manner, apps can help reduce the burden on hospitals, provide access to credible information, track the symptoms and mental health of individuals, and discover new predictors [25]. However, the security and privacy of the shared data still need to be improved. An analysis of 50 mobile apps developed worldwide during the COVID-19 pandemic indicated that only 16 of them guaranteed that users’ data would be anonymous, encrypted, and secured, and also would be transmitted on the web and reported only in an aggregated format. These apps continuously collect and process sensitive personally identifiable information, such as health information, location, and direct identifiers. The fear of having their data used in the wrong way could decrease users’ adherence to this type of app [26]. The project Conecte SUS implies the circulation of patient data between mobile apps and digital platforms. As the digital health applications are executed in a heterogeneous and decentralized environment, blockchain technology was adopted at the RNDS because it presents itself as the most robust solution to security issues, naturally addressing security, performance, access, and scalability issues. Recent studies have shown the advantages of using blockchain technology to register health data [27-29]. The technology guarantees information security and allows a distributed location, maintaining the local access of each health provider to their data and sharing through an interconnected blockchain network between participating organizations [28,30].

To ensure that data will be collected and used safely and transparently, the Brazilian government sentenced the General Data Protection Law (*Lei Geral de Proteção de Dados Pessoais*, LGPD) on August 16, 2020. The main objective is to make digital health an innovative path capable of promoting the service’s improvement to the population and the transition and continuity of care through safe and transparent access to the clinical history of the user. The LGPD defines people as the exclusive owners of their data and determines the health data as sensitive data. The use of personal health data by third parties will not be allowed. However, disclosing certain information for the benefit of the community or public health reasons is permitted, without prejudice to the patient's intimacy and privacy, through anonymity. Individual health data can only be accessed by health professionals involved in the case (through the Conecte SUS portal) and with the prior consent of the data owner, who must be informed both about the use and who made that use and its purpose.

After the end of the implementation phase, only with COVID-19 data, the RNDS will resume its initial strategy, prioritizing establishments that already use the Electronic Citizen Medical Record system and establishments that use the Management Application for University Hospitals. The Electronic Citizen Medical Record is the service that allows the collection of health records. It is available free of charge to municipal governments for the management of primary health care. The new version of this service and the RNDS are at an advanced stage in the 10 municipalities chosen for this initial phase [31].

The adoption of EHRs has provided consolidated technology for storing patient clinical data [32]. This concept has more recently evolved into the idea of the personal electronic health record (PHR). The main difference between a traditional RES and a PHR is that the latter allows interaction with patients through access to clinical data [33]. It is expected that Conecte SUS will be the central point of access to procedures performed in the future. The tool should function as a PHR and allow citizens to monitor their health situation, enable health professionals to access their patients’ tests and clinical history to continue care, and allow managers to monitor health indicators.

A long road has been taken to facilitate the exchange of health information between different primary care establishments in Brazil. Strategies that are part of the digital health revolution in Brazil were redirected to mitigate the damage caused by SARS-CoV-2. Still, these strategies must resume their purposes when the situation becomes stable. The ethical use of health data through an information platform that has high availability, but is safe and accessible at the same time, will undoubtedly allow greater participation by society. The emergence of new services, research, and innovation will benefit the population and Brazil.

## Conclusion

Brazil has been working to digitally transform the health sector since the Brazilian digital health strategy launch in 2017. The COVID-19 pandemic accelerated this transformation and has created enormous challenges for decision makers. The pandemic is a national test of acceptance and of the ability of Brazilian citizens to use and engage with digitizing health and communications services. It is still too early to assess Brazil’s experience in implementing digital solutions for the entire population; there is a long way to go before achieving digital health implementation due to technological issues and territorial, financial, and ethical issues. The approaches of IT and health professionals are also fundamental because the digital health revolution will only occur if clinicians embrace this challenge. More research is needed to explore and analyze the pitfalls, make the right decisions, and define the challenges of this digital experience for the unified health system, private institutions, and employees and consumers.

## Abbreviations

CAPES: Coordenação de Aperfeiçoamento de Pessoal de Nível Superior
EHR: electronic health record
FHIR: Fast Healthcare Interoperability
ICT: information and communications technology
Informatiza APS: Programa de Apoio à Informatização e Qualificação dos Dados da Atenção Primária à Saúde (Program to Support Computerization and Qualification of Primary Health Care Data)
IT: information technology
LGPD: Lei Geral de Proteção de Dados Pessoais (General Data Protection Law)
PHR: personal electronic health record
PROADI-SUS: Programa de Apoio ao Desenvolvimento Institucional do Sistema Único de Saúde (Institutional Development Support Program of the Unified Health System)
RNDS: Rede Nacional de Dados em Saúde (National Health Data Network)
SARS: severe acute respiratory syndrome
SIVEP-Gripe: Sistema de Informação de Vigilância Epidemiológica da Gripe
SUS: Sistema Único de Saúde (Unified Health System)
WHO: World Health Organization
